# Characterization of *Gfat1* (*zeppelin*) and *Gfat2*, Essential Paralogous Genes Which Encode the Enzymes That Catalyze the Rate-Limiting Step in the Hexosamine Biosynthetic Pathway in *Drosophila melanogaster*

**DOI:** 10.3390/cells11030448

**Published:** 2022-01-27

**Authors:** Shawn Cotsworth, Catherine J. Jackson, Graham Hallson, Kathleen A. Fitzpatrick, Monika Syrzycka, Alistair B. Coulthard, Amy Bejsovec, Marcella Marchetti, Sergio Pimpinelli, Simon J. H. Wang, Robert G. Camfield, Esther M. Verheyen, Donald A. Sinclair, Barry M. Honda, Arthur J. Hilliker

**Affiliations:** 1 Department of Molecular Biology and Biochemistry (MBB), Simon Fraser University, 8888 University Dr., Burnaby, BC V5A 1S6, Canada; scotswor@gmail.com (S.C.); catherinejoanjackson@gmail.com (C.J.J.); gdhallson@gmail.com (G.H.); kathleef@sfu.ca (K.A.F.); monika.syrzycka@abbvie.com (M.S.); simon_wang@sfu.ca (S.J.H.W.); everheye@sfu.ca (E.M.V.); don_sinclair@sfu.ca (D.A.S.); honda@sfu.ca (B.M.H.); 2 Department of Plastic and Reconstructive Surgery, Institute for Surgical Research, University of Oslo, N-0424 Oslo, Norway; 3 The Department of Medical Biochemistry, Oslo University Hospital, N-0424 Oslo, Norway; 4 Institute of Oral Biology, Faculty of Dentistry, University of Oslo, N-0424 Oslo, Norway; 5 Allergan Canada, 500-85 Enterprise Blvd, Markham, ON L6G 0B5, Canada; 6 Department of Biology, York University, Toronto, ON M3J 1P3, Canada; alistair@yorku.ca; 7 Department of Biology, Duke University, Durham, NC 27708, USA; bejsovec@duke.edu; 8 Department of Biology and Biotechnology “C. Darwin”, “Sapienza” University of Rome, 00185 Rome, Italy; marcella.marchetti@uniroma1.it (M.M.); sergio.pimpinelli@uniroma1.it (S.P.); 9 BC Genome Science Centre, 675 West 10th Avenue, Vancouver, BC V5Z 1L3, Canada; rcamfield689@gmail.com

**Keywords:** *Gfat1*, *Gfat2*, HBP, hexosamine biosynthesis, *Drosophila heterochromatin*, *zeppelin*

## Abstract

The *zeppelin* (*zep*) locus is known for its essential role in the development of the embryonic cuticle of *Drosophila melanogaster*. We show here that *zep* encodes *Gfat1* (*Glutamine: Fructose-6-Phosphate Aminotransferase 1*; *CG12449*), the enzyme that catalyzes the rate-limiting step in the hexosamine biosynthesis pathway (HBP). This conserved pathway diverts 2%–5% of cellular glucose from glycolysis and is a nexus of sugar (fructose-6-phosphate), amino acid (glutamine), fatty acid [acetyl-coenzymeA (CoA)], and nucleotide/energy (UDP) metabolism. We also describe the isolation and characterization of lethal mutants in the euchromatic paralog, *Gfat2* (*CG1345*), and demonstrate that ubiquitous expression of *Gfat1^+^* or *Gfat2^+^* transgenes can rescue lethal mutations in either gene. *Gfat1* and *Gfat2* show differences in mRNA and protein expression during embryogenesis and in essential tissue-specific requirements for Gfat1 and Gfat2, suggesting a degree of functional evolutionary divergence. An evolutionary, cytogenetic analysis of the two genes in six *Drosophila* species revealed *Gfat2* to be located within euchromatin in all six species. *Gfat1* localizes to heterochromatin in three melanogaster-group species, and to euchromatin in the more distantly related species. We have also found that the pattern of flanking-gene microsynteny is highly conserved for *Gfat1* and somewhat less conserved for *Gfat2*.

## 1. Introduction

**Figure 1:**
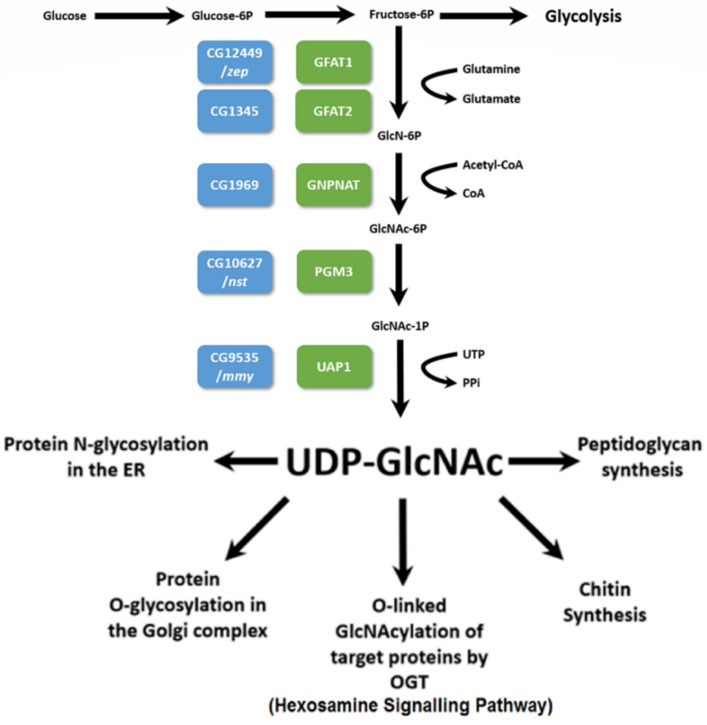
Schematic depiction of the Hexosamine Biosynthetic Pathway (HBP) showing the key metabolic inputs, the reaction steps, along with the enzymes/genes responsible for catalysis and the important outputs. Blue boxes correspond to the gene names in *Drosophila melanogaster* and green boxes correspond to the enzymes that they encode (see the Introduction). Modified from [3,4].

The HBP diverts fructose-6-phosphate from glycolysis to generate UDP-N-acetylglucosamine, an important precursor used in the formation of glycoproteins (e.g., GlcNAcylation by OGT, *O*-GlcNAc transferase), proteoglycans, and other important biomolecules (Figure 1) [1,2,3,4]. The rate-limiting step in the pathway is catalyzed by Glutamine-fructose-6-phosphate transaminase 1 or GFPT1 in humans (hereafter called hGfat1) and Gfat1 in Drosophila, and Glutamine-fructose-6-phosphate transaminase 2 or GFPT2 in humans (hereafter called hGfat2) and Gfat2 in Drosophila. In Drosophila, *C*. *elegans*, mice, and humans, Gfat1 and Gfat2 are encoded by two separate genes, and this may also be the case for *Aedes aegypti* [5,6,7,8]. In contrast, it appears that there is only a single *Gfat* gene/enzyme in fungi [9,10,11]. The distinction between hGfat1 and hGfat2 is the presence of two putative Protein Kinase A (PKA) sites in hGfat1, but only a single site in hGfat2 [5,12,13]. In *Drosophila melanogaster*, one of the PKA sites is conserved in both Gfat1 and Gfat2. Although the other site is partially conserved (albeit presumably non-functional) in Gfat1, it is absent from Gfat2 [5].

Gfat enzymes convert fructose-6-phosphate and glutamine to glucosamine-6-phosphate (GlcN-6P) and glutamate (Figure 1). The GlcN-6P is then rapidly processed by a series of other enzymes in the HBP, ultimately generating the final product UDP-GlcNAc, an essential substrate for a variety of highly conserved cellular and organismal functions [4,6]. These include (1) N-glycosylation; (2) *O*-linked glycosylation (e.g., mucin-type O-glycosylation); (3) *O*-linked GlcNAcylation, an extremely important post-translational modification of serine and threonine residues of target proteins catalyzed by *O*-GlcNAc transferase (OGT); and (4) the formation of structural biomolecules such as chitin, a long chain GlcNAc polymer that is one of the most abundant macromolecules found in nature and is the primary component of the arthropod cuticle/peritrophic matrix [6,14].

Given the multifaceted cellular requirements for the HBP, it is not surprising that suboptimal functioning of the pathway has been linked to a wide variety of diseases, including heart disease, diabetes, neurodegenerative diseases, cellular stress diseases, premature aging, and cancer [1,2,3,6,15,16,17,18,19]. Many of these diseases stem from aberrant *O*-linked GlcNAcylation [20,21].

Since chitin is a major component of the insect cuticle [22] and because the end product of the HBP serves as the substrate for chitin synthesis, a reasonable prediction is that genetic defects in HBP enzymes will block chitin synthesis and, thus, cuticle production in flies. Indeed, this is true for *mummy* (*mmy*), which encodes UDP-N-acetylglucosamine diphosphorylase (UAP1), the enzyme that catalyzes the final step in the HBP (Figure 1). Hypomorphic *mmy* alleles exhibit a bloated embryonic cuticle, while null alleles fail to secrete an embryonic cuticle [23]. In an earlier study, workers determined that devitellinized embryos homozygous for a mutant allele of a previously unknown gene called *zeppelin* (*zep*) also displayed an expanded embryonic cuticle, which was dubbed the “blimp” phenotype [24]. The *zep* locus was mapped to 3R pericentric heterochromatin (3R het). Interestingly, *Gfat1* has been previously mapped to 81F in 3R het [5].

The notorious silencing properties of centromeric heterochromatin with respect to relocated euchromatic genes, coupled with the paradoxical finding that heterochromatic genes require a heterochromatic environment for optimal expression (reviewed in [25]), have long intrigued Drosophila researchers. Hence, Hilliker and Honda have participated in a collaboration aimed at contributing to the characterization of heterochromatin in Drosophila, with the view to obtaining more insight into the relationship between chromatin structure and gene expression, as well as the evolution of heterochromatin. Much of our work has focused on correlating genetically identified loci with existing gene models in autosomal heterochromatin in *Drosophila melanogaster* e.g., see [25,26]. In the current work, we show that the essential *zep* locus corresponds to the *Gfat1* gene. Furthermore, we have expanded our analysis by exploring the relationship between the *Gfat1* and *Gfat2* genes and their products. Thus, we describe the isolation and characterization of lethal mutants in the euchromatic paralog, *Gfat2,* and show that ubiquitously expressed *Gfat1*^+^ and *Gfat2*^+^ transgenes can rescue lethal alleles in either gene, indicating that the two enzymes are functionally equivalent in *D*. *melanogaster*. In addition, our RT-qPCR analysis and the available RNAseq and proteomic data reveal distinct embryonic expression patterns for the two genes. We also provide evidence that while there are essential requirements for Gfat2 in the nervous system and muscles, this does not appear to be the case for Gfat1. This essential tissue-specificity of Gfat2 is likely due, at least in part, to the need to provide UDP-GlcNAc for essential functions of OGT. In contrast, our data show that both Gfat1 and Gfat2 have essential functions in the trachea.

We have also carried out an evolutionary cytogenetic analysis to determine where the two paralogues are located in five other Drosophila species. The *Gfat2* gene is located in euchromatin in *D*. *melanogaster* and all five other species examined. On the other hand, the *Gfat1* gene is located in heterochromatin in *Drosophila melanogaster* and the two other species in the melanogaster group but it is located in euchromatin in the three more distantly related species. Finally, we report microsynteny data for genes that immediately flank *Gfat1* and *Gfat2* in the six species. These data show that the gene arrangement in *D*. *melanogaster* is completely conserved for *Gfat1* and mostly conserved for *Gfat2*. The *Gfat1* data suggest that the evolutionarily recent acquisition of a hetereochromatic environment by this gene occurred by the relocation of a genomic block containing several genes, a finding similar to that reported for the *light* gene and its neighbours [27].

## 2. Materials and Methods

### 2.1. Pre-Existing Drosophila Stocks, Routine Culture Conditions, Identification of New Zep/Gfat1 Alleles and Routine Genetic Crosses

Stock centre transgenic RNAi lines used in the present study are described in Table S1. It is noteworthy that both v24539 and B42892 target all nine identified *Gfat1* transcripts that are listed at Flybase ([28] and data not shown). Most other stocks used were either acquired from the Bloomington Drosophila stock centre (BDSC) or in some cases, directly from research groups. The generation/origin of *Gfat1* RNAi and *Gfat1^+^* transgenes is described below. Standard Drosophila medium was used throughout this study. Fly stocks were maintained at 25 °C or 18 °C and most experiments were conducted in vials at 25 °C or 29 °C. New EMS-induced *Gfat1*/*zep* mutant alleles were identified from two sources via non-complementation with the original *zep^LP13^* allele. Five alleles, each designated with a z superscript and identifying number, were identified among a collection of third chromosome recessive lethal lines (each was marked with *st* and *ry*) kindly provided by the Zuker lab [29]. Two additional alleles, *I400#1* and *I400#* were among several third chromosome recessive lethal lines (each marked with *th st cp in ri p^p^*) kindly provided by Dr. M. Leptin [30]. *Df(3R)10*-65, *kni^ri−1^ p^p^*/*TM3*, *Ser* (BDSC #2597) and *Df(3R)4-75*, *kni^ri−1^ p^p^*/*TM3*, *Ser* (BDSC #2598) were generated previously by Marchant and Holm [31,32]. More recently, it has been determined that *Df(3R)4-75* is a multi-breakpoint pericentric inversion [33,34]. *w^1118^*; *Df(3R)BSC460*/*TM6C*, *Sb cu* (BDSC stock#24964) and w^1118^; *Df(3R)BSC567*/*TM6C*, *Sb* (BDSC stock#25390) are two deletions that uncover *Gfat2*. Four additional deletions that uncover *zep* lethality and delete *Gfat1* were also used in the current study: *Df(3R)8740#20* (also called *zep^8740#20^*), *Df(3R)8740#22* (also called *zep^8740#22^*), and *Df(3R)EP-167* (also called *zep^167^*), were recovered in a P-element-induced male recombination study, and *Df(3R)7B-90, e* was X-ray-induced [35,36].

*Inter se* complementation analysis involving all pairwise combinations of the eight *zep* alleles was carried out at 25 °C, scoring a minimum of 100 emerging adult progeny per cross. The RNAi crosses were performed at 29 °C. Six of ten different *UAS*-*RNAi* transgenes for *Gfat1* generated in this study (see below) were tested for viability using either of the strong ubiquitously-expressing drivers, *Tub-GAL4*/*TM3, Sb*, or *Act5C-GAL4*/*CyO*, and all were lethal (Table S2). A heat-shock *GAL4* driver was also used to express specific *Gfat1* RNAi transgenes with a view to identifying adult phenotypes. The btl-GAL4 experiments were completed later than the others using an incubator set at 30 °C.

### 2.2. Cuticle Analysis of Newly-Isolated Zep Alleles

Embryonic cuticle preparations to test the new *zep* alleles for the blimp phenotype were carried out as described in [24].

### 2.3. Sequence Analysis of Zep Alleles

Genomic DNA corresponding to 500 bp segments spanning the entire coding sequence plus the exon/intron splice junctions of the *Gfat1* gene was isolated from embryos homozygous for each of the eight *zep* alleles by PCR using the primers listed in Table S3. The resulting DNA segments were then subjected to standard DNA sequence analysis by Macrogen Inc. (Seoul, Korea; http://dna.macrogen.com/eng/ (accessed on 29 December 2021)). The sequences were then compared to the Flybase [28] genomic sequence using BLASTN to identify point mutations and BLASTP to identify changes at the amino acid level.

### 2.4. Isolation and Genetic and Molecular Characterization of Putative Gfat2 Excision Mutations

The *Gfat2* gene is located in euchromatin on the right arm of chromosome 3 at cytological position 98C4 [28]. We generated lethal excisions by using *Sb* Δ *2.3* to mobilize a P-element located in the 5′ UTR of the gene, *w*^67c23^ *P{EPgy2}Gfat2^EY21762^* (or *EY21762*: BDSC #22502) [28], selecting for *w^−^* excisions and then testing each for recessive lethality. *w*^−^/*TM6*, *Sb* males from each of the lethal lines were crossed separately to either *Df(3R)BSC460*/*TM6, Tb*, or *Df(3R)BSC567*/*TM6, Tb* in order to confirm that the lethality mapped to the segment containing the *Gfat2* gene. Putative excision mutations that were lethal in combination with either deletion were re-balanced with *TM3, Sb Ser e^s^ twiGAL4-EGFP*, and each was tested for complementation with w*; *PBac{GAL4D,EYFP}larp^43^*/*TM3*, *Sb* (BDSC #78330), a lethal allele of the *La*-*related protein* (*larp*) gene, which lies immediately proximal to *Gfat2* [28]. Since there were no available lethal mutations in the immediately distal gene, *Moca*-cyp, DNA from single embryos homozygous for each of putative excision lesions that complemented the *larp* allele, was separately subjected to PCR analysis in order to identify molecular excisions that did not extend into *Moca*-*cyp*. The *Moca*-*cyp* primers used for this purpose were: forward primer 5′-AGTTCTGAGTAGAGCTGGCAACGCC-3′ and reverse primer 5′-ACAGCAGCACACACACACAAGCG-3′.

### 2.5. Sequence Analysis of Gfat2 Mutants

DNA from homozygotes for each of the two *Gfat2*-specific excision mutants was subjected to sequence analysis as follows: The entire gene was PCR amplified from single homozygous embryos using primers that annealed to segments flanking the coding region. Each PCR product was blunt-end ligated into the Fermentas pJET 1.2 vector and sent to UBC-NAPS for DNA sequencing using the forward primer 5′- GCGCCGTTCACTTGTCTTGTCAAT-3′ and the reverse primer 5′-TCACACCCTTGTACTGCAGCTTCT-3′, and the sequence data obtained were compared to the wild type *Gfat2* sequence [28].

### 2.6. Lethal Phase Analysis of a Gfat2 Deletion Mutant

*Gfat2^10A−2^/TM3 Sb Ser twi 2x-eGFP* virgin females were crossed to *Df(3R) BSC460/TM3 Sb Ser twi 2X-eGFP* males and incubated at 25 °C for 5 days. The cross was then caged over an embryo lay plate for 6 h at 25 °C after which adult flies were removed and the plate incubated overnight. *Gfat2^10A−2^/Df(3R) BSC460* embryos that did not fluoresce under examination by standard GFP fluorescence microscopy were placed on a new plate and examined the following day. The number of unhatched embryos and first instar larvae (L1) remaining on the plate was used to determine the number of organisms that died during embryogenesis. The surviving larvae were counted again the following day.

### 2.7. Generation of a Gfat1^+^ Rescue cDNA Construct

We obtained the RE72989 EST with cDNA corresponding to *Gfat1*-RA, inserted it into the pBluescript shuttle vector and ultimately cloned it into pUAST [36,37]. This construct was sequenced by Macrogen Inc. and then sent to BestGene Inc. (2140 Grand Ave, Chino Hills, CA, USA) for the generation of transgenic lines.

### 2.8. Gfat1 and Gfat2 Rescue Crosses

The test for genetic rescue of *zep* mutants by a ubiquitously expressed *UAS*-*Gfat1*^+^ cDNA was performed by crossing *UAS*-*Gfat1^+^/CyRoi; Gfat1^LP13^/TM3, Sb* males to *Actin5C-GAL4/CyO; Df(3R)7B-90, e/TM3, Sb* females. The test for rescue analysis of *Gfat2* excision mutants by a *UAS*-*Gfat1^+^* cDNA was performed by crossing *UAS*-*Gfat1^+^*/*CyRoi; Gfat2^10A−2^/TM3*, *Ser* males to *Actin5C-GAL4/CyO; Df(3R)567/TM3*, *Ser* females. Finally, a *UAS*-*Gfat2^+^* cDNA transgenic line (generously provided by Dr. L. Partridge) was tested for the rescue of *Gfat1* and *Gfat2* mutants in the following crosses: (i) *UAS*-*Gfat2^+^*/*Cy, Roi*; *Df(3R)BSC460*/*TM6B, Tb* females and *Actin5C-GAL4*/*CyO*; *Gfat2^10A−2^*/*TM3*, *Ser* males; (ii) *UAS*-*Gfat2^+^*/*CyRoi*; *Gfat1^z−1904^*/*TM6 B, Tb* females, and *Actin5C-GAL4*/*CyO*; *Gfat1^I400#8^*/*TM3, Ser* males [38]. In all rescue tests, all surviving progeny of the diagnostic crosses were classified and counted.

### 2.9. Gfat1 RNAi Stocks and Crosses

The *Gfat1* RNAi stocks were generated by amplifying a 290 bp fragment from the RE72989 cDNA clone using the forward primer 5′-GACTCCTTCCTCGAGCTGT-3′ and the reverse primer 5′-TCAGAATTCCTTTCCGAACGC-3′. Underlined base pairs were altered from the known *Gfat1* sequence ([28] in order to create artificial *Xho*I and *Eco*RI restriction sites in the forward and reverse primers, respectively [28]. The 290 bp amplicon was ligated into the pTZ57R shuttle vector then digested with *Xho*I and *Eco*RI and inserted into the pSympUast vector. Ten transgenic strains containing the *Gfat1* RNAi construct were generated in the *w*^1118^ background by BestGene Inc. (2140 Grand Ave, Chino Hills, CA, USA).

### 2.10. Real-Time qPCR

RNA was isolated from wild type *Oregon-R* embryos (synchronized at specific time intervals after egg-lay) using TRIZOL. One μg of each RNA sample was treated with DNAseI for 1 h at 37 °C and 364 ng of DNA free RNA from each sample was used to create cDNAs using the BioRad**™** iScript select cDNA synthesis kit using its random primers. qPCR primers specific for amplifying *RpL32, Gfat1*, and *Gfat2* were obtained from IDT along with a probe possessing a 5′ 6-FAM fluorophore, a 3′ Iowa Black FQ quencher, and an intermediate ZEN quencher located 9bp from the 5′end of the probe that would anneal to each individual amplicon (see Table S3 for qPCR primer sequences). Each qPCR reaction was prepared using 2 µL of the cDNA sample, 1 µL of a cocktail consisting of the primers and the fluorophore probe, 10 µL of BioRad**™** iTaq Supermix, and 7 µL of ddH_2_O and cycled using an Applied Biosystems**™** Step-One Real-Time PCR System Machine. Standard curves were generated using each primer set in order to determine PCR efficiency. A relative quantification of *Gfat1* and *Gfat2* expression at each of these stages was determined relative to the 0–90 min sample according to the method described in [39].

### 2.11. Tests for Essential Tissue-Specific Requirements for Gfat1 and Gfat2

In order to test for possible essential requirements for Gfat1 and Gfat2 in specific tissues, the effects of knockdown of their respective genes in the nervous system and muscles using various RNAi transgenes for *Gfat1* and *Gfat2* were examined as follows: Males bearing individual *Gfat1* or *Gfat2* RNAi transgenes were mated to females bearing the larval pan-neural driver *Appl-GAL4* (generously provided by Dr. U. Pandey) or the muscle driver *Mef2-GAL4*, and the offspring were raised at 29 °C. Since the product of the HBP is used as the substrate for GlcNAcylation by *Ogt*/*sxc* (*CG10392*), analogous crosses were carried out using a TRiP *Ogt* RNAi line. In a subsequent experiment, to test for essential requirements of Gfat1 and Gfat2 in tracheae, males bearing specific *Gfat1* or *Gfat2* RNAi transgenes were mated to females bearing the trachea driver *breathless* (*btl*)-*GAL4*, and the offspring were raised in an incubator set at 30 °C. In each case, adult survival associated with specific RNAi-induced knockdown was assessed and knockdown was deemed to be lethal if no diagnostic adult offspring were observed in comparison to the survival of a minimum of 60 internal control adults from the same cross. Where relevant, the designations of male semi-lethality or weak semi-lethality are explained in the table footings.

### 2.12. Fluorescence In Situ Hybridization (FISH) Localization of Gfat1 and Gfat2 in Different Species of Drosophila

Polytene and mitotic in situ analyses were carried out as described previously [40]. Probes were differentially labeled by nick translation with digoxigenin- or biotin-coupled dUTP, and, after hybridization at 37 °C overnight, the signal was detected with a fluorescein avidin or antidigoxigenin–rhodamine antibody. DNA was counterstained with DAPI before image capture. Microsynteny analysis was performed using species data available at FB2017_05 (Dmel Release 6.18) from http://www.flybase.org and/or https://blast.ncbi.nlm.nih.gov/Blast.cgi (accessed on 29 December 2021). 

## 3. Results

### 3.1. The Zep Locus Corresponds to Gfat1

The *zep* gene was originally mapped to 3R het based on non-complementation between *zep^LP13^* and *Df(3R)4-75* [24]. Since both *mmy* and *zep^LP13^* mutants exhibit expanded embryo phenotypes and because *mmy* encodes an HBP enzyme, a reasonable hypothesis is that the 3R het gene *Gfat1* [5] corresponds to the *zep* locus. This hypothesis is strongly supported by the finding that *zep* is the only essential gene deleted by *Df(3R)8740#20* and *Df(3R)8740#22*, plus the fact that both deficiencies also remove *Gfat1* (Figure S1) [35,36,41]. The fact that the *Gfat1* insertion allele, y w^*^; Mi{MIC}Gfat1^MI11277^/TM3, Sb Ser (BDSC 56582), failed to complement *zep^LP13^* (data not shown) is further evidence that the *zep* locus corresponds to the *Gfat1* gene.

**Figure 2:**
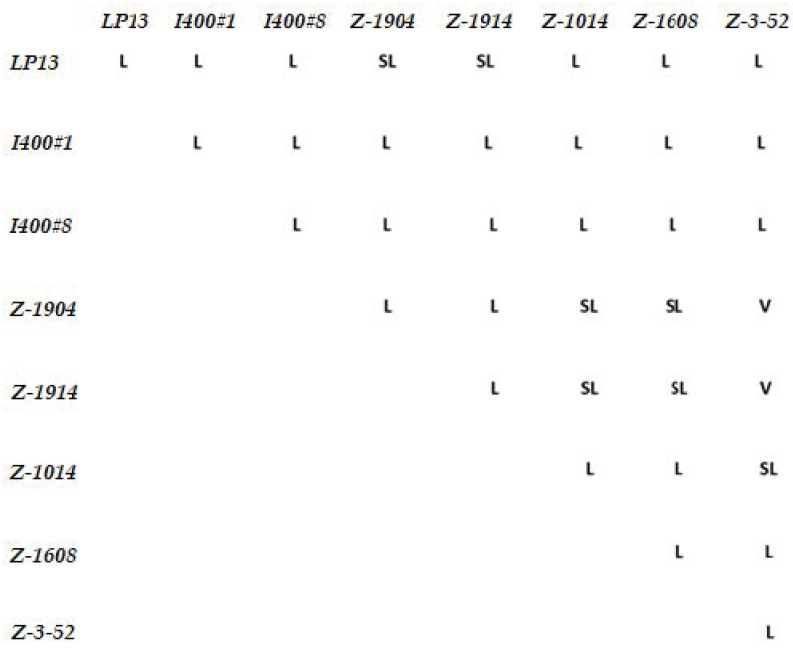
Results of inter se complementation analysis of zep alleles. The mutant third chromosomes were balanced with either TM3, Sb Ser, or TM3 Ser. A minimum of 100 progeny per cross was examined. L = lethal; SL = semi-lethal (less than 50% of expected transheterozygous or homozygous progeny relative to balancer progeny); V = viable.

We extended our analysis by isolating and characterizing seven new *zep* alleles based on the failure to complement *zep^LP13^* from a large collection of EMS-induced recessive lethal mutations provided by the Zuker and Leptin groups [29,30]. The *inter se* complementation data for the eight *zep* alleles are shown in Figure 2. Most pairwise combinations were lethal. However, several combinations, involving transheterozyotes between *zep^z1904^*, *zep^z1914^*, or *zep^3−52^* and various other alleles, were either semi-lethal (less than 50% of expected progeny) or viable, suggesting that these may be hypomorphic *zep* alleles. Indeed, the weakest alleles, *zep^z1904^* and *zep^z1914^*, exhibited the same complementation pattern, and they are semi-lethal in combination with *Gfat1* deletions (data not shown). Interestingly, many surviving transheterozygotes had extended legs with melanin deposits at the joints. This phenotype, which was also observed for some survivors when a *Gfat1-RNAi* transgene was expressed continuously at 29 °C using a heat-shock *GAL4* transgene (Figure S2), resembles the effects of reduced expression of the *Splayed* locus, a putatively haplo-abnormal gene positioned in 81F-82A [42,43]. Thus, it is possible that the *Gfat1*/*zep* is allelic to *Spl*.

**Figure 3:**
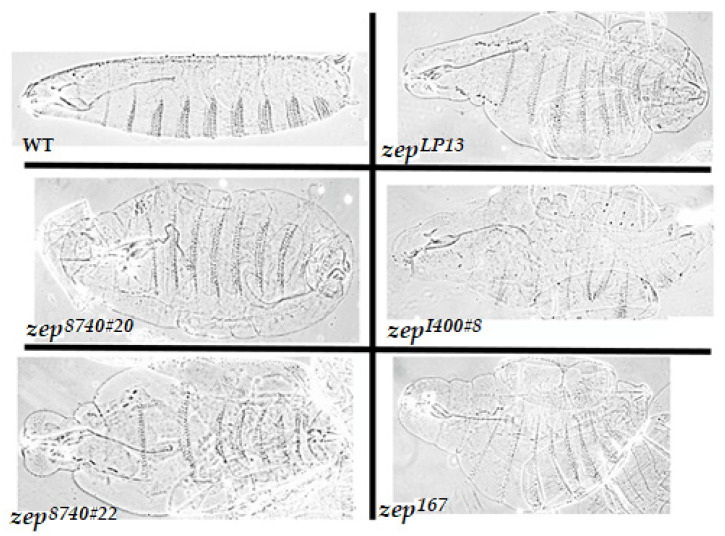
Expanded cuticle phenotypes of the strongest *zep* alleles. *zep^LP13^* and *zep^I400#8^* contain stop codons (see Figure 4) whereas *zep^8740#20^*, *zep^8740#22^*, and *zep^167^* delete *Gfat1* plus two or more flanking genes (see Figure S1). A wildtype (Oregon-R) embryo is shown in the top left panel. Homozygous mutant and wildtype embryos were prepared as described in Ostrowski et al. [24] The embryos are oriented with anterior to the left and posterior to the right. Note the pronounced bloating of the mutant embryos relative to wildtype.

In order to test whether the newly isolated *zep* alleles also exhibited the blimp phenotype, cuticle preparations of mechanically devitellinized homozygous mutant embryos were performed as previously described [24]. Indeed, embryos homozygous for each of the seven new EMS *zep* alleles and three *Gfat1* deletions exhibit the diagnostic expanded cuticle phenotype to varying degrees that correlate with allele severity (Figure 3 and data not shown), consistent with the hypothesis that the *zep* locus is *Gfat1*.

**Figure 4:**
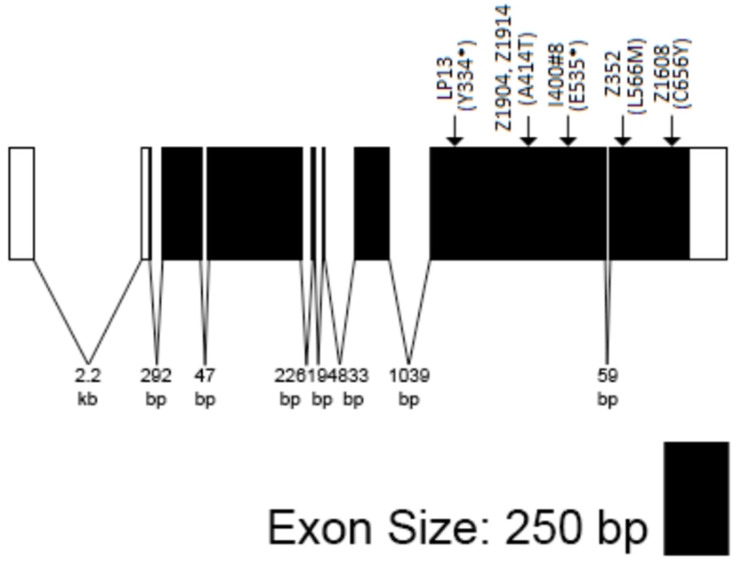
Transcript model of *Gfat1-RN*. Locations of mutations are given above and are marked by arrows along the gene model. *zep^LP13^* and *zep^I400#8^* contain stop codons (indicated by asterisks) after Tyr334 and Glu535, respectively; *zep^z1904^ and zep^z1914^* contain the identical missense mutation, A414T; *zep^3−52^* contains the missense mutation, L566M; and *zep^z1608^* contains the missense mutation, C656Y.

Sequence data confirmed that *Gfat1* and *zep* are the same genes (summarized in Figure 4). A total of Six of the *zep* alleles possess either a nonsense or missense mutation in their coding sequence. Thus, *zep^LP13^* and *zep^1400#8^* contain premature stop codons: *zep^LP13^*: Tyr334*, and *zep^I400#8^*: Gln535* presumably results in truncated proteins, each possessing the Glutaminase domain, but lacking one or both Isomerase domains. In contrast, each of the other four alleles contains a missense mutation: *zep^3−52^*: L588M; *zep^z1608^*: C656Y; *zep^z1904^*, and *zep^z1914^* contain the identical lesion: A414T. Each of these represents a substitution of a conserved residue in one of the Isomerase domains. However, based on the complementation data (see above), the *zep^z1904^*, *zep^z1914^*, and *zep^3−52^* lesions do not appear to block *Gfat1* activity completely. The remaining two alleles, *zep^z1014^* and *zep^I400#1^*, have no non-polymorphic changes in their protein-coding or exon/intron junction sequences.

### 3.2. Isolation, Sequence and Lethal Phase Analysis of Gfat2 Mutant Alleles

**Figure 5:**
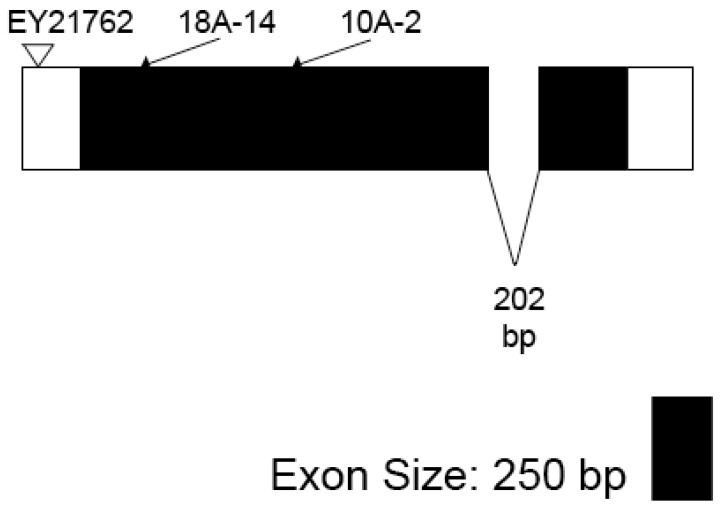
Transcript model of *Gfat2*-*RA*. The approximate location of the P-element insert used for the excision experiment is shown above by an inverted triangle. The approximate end points for the excision deletions *Gfat2^18A−14^* (498 bp in length) and *Gfat2^10A−2^* (1038 bp in length) are shown above by arrows.

We identified several putative *Gfat2* lesions from the P-element-excision study described in the Materials and Methods. A total of thirty-eight *w^−^* excisions were recessive lethal and nine of these that were lethal with one of the Gfat2 deletions, *Df(3R)BSC460*/*TM6, Tb* or *Df(3R)BSC567*/*TM6, Tb* were viable in combination with *larp^43^.* All nine were subjected to PCR analysis to check for genomic integrity of *Moca-cyp* as described. This analysis identified three lesions for which *Moca-cyp* was intact: *1C-25*, *10A-2*, and *18A-14* [44]. Sequence analysis of two of these, designated as *Gfat2^10A−2^* and *Gfat2^18A−14^*, revealed that each involved a *Gfat2*-specific deletion (Figure 5), thereby confirming that the gene is essential. The Gfat210A-2 mutant is a deletion beginning 50 bp from the 5′ end in the 5′UTR, extending 1038 bp towards the 3′ end, deleting part of exon 1 and all of the glutaminase domain. The Gfat218A-14 mutant is a deletion beginning 50 bp from the 5′ end of the 5′UTR and extending 498 bp towards the 3′ end, deleting part of exon 1 and just under a third of the glutaminase domain. Both deletions retained the 5′-CATGATGAAATAA-3′ sequence that was originally part of the terminal repeat of the P-element in the EY21762 line. Lethal phase analysis of 100 Gfat210-A2/Df(3R)BSC460 hemizygotes revealed a biphasic pattern (Table 1): 59% died during embryogenesis and 41% survived to the L1 stage. The Gfat2- L1 larvae failed to grow and died shortly after hatching. Interestingly, the dead Gfat2-embryos displayed no obvious cuticle phenotype (data not shown). This contrasts with the embryonic lethal phase and blimp phenotypes of zep/Gfat1 alleles.

### 3.3. Gfat1/Zep and Gfat2 Mutants Can Be Rescued by Ubiquitous Expression of Gfat1^+^ and Gfat2^+^ cDNA Transgenes

Rescue analysis also confirmed that *Gfat1* and *zep* are the same genes. We were able to rescue the lethality of a *zep*/*Gfat1* allele in combination with *Df(3R)7B-90e* by using a *UAS*-*Gfat1*^+^ cDNA under the control of *Act5C-GAL4* (top panel, Table 2). Furthermore, constitutive expression of the *UAS-Gfat1^+^* allowed the rescue of flies hemizygous for either *Gfat2^10A−2^* or *Gfat2^18A−14^* (second panel in Table 2). Finally, both *Gfat1* and *Gfat2* mutant alleles were rescued by constitutive expression of the *UAS*-*Gfat2^+^* transgene (bottom two panels, Table 2). Together, these data suggest that the two Gfat enzymes are functionally equivalent.

### 3.4. Gfat1 and Gfat2 Genes Exhibit Different Expression Patterns during Development

**Figure 6:**
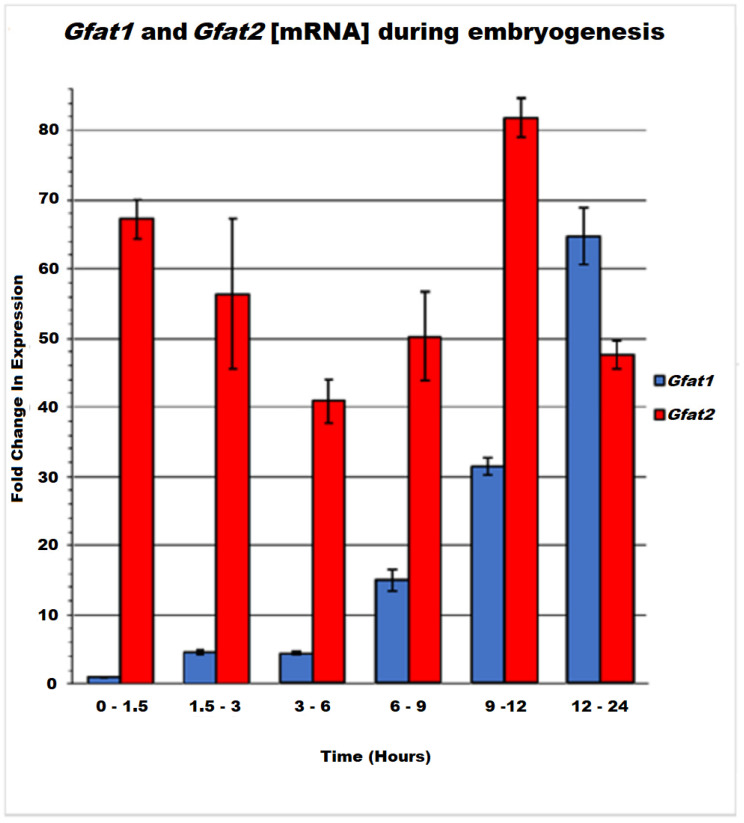
RT-qPCR analysis of Gfat1 and Gfat2 mRNA using RpL32 as a reference gene and the Gfat1 0–1.5 h sample as the control group, at different stages of embryogenesis.

The different lethal phases and embryonic phenotypes of *Gfat1* and *Gfat2* mutants raised the possibility that Gfat1 and Gfat2 may have somewhat different physiological roles in the fly. We investigated this possibility initially by profiling the mRNA pattern of each gene at specific intervals during embryogenesis using RT-qPCR analysis (Figure 6). Our data show that *Gfat2* mRNA is relatively abundant throughout embryogenesis, with the highest level observed at 9–12 h after egg-lay (AEL). Furthermore, perceptible expression of the gene within 0–1.5 h AEL indicates the significant maternal contribution of the *Gfat2* mRNA. In contrast, *Gfat1* mRNA levels were relatively low until 6–9 h AEL, reaching a peak near the end of embryogenesis, which coincides with the time of embryonic cuticle deposition [24]. These mRNA patterns are similar but not identical to those revealed by the more refined modENCODE Refseq data [28]. These show moderately high expression of *Gfat2* throughout most embryogenesis, with high expression in the 8–12 h (~stages 12–15) and 14–16 h AEL (~stage 16) intervals. Similar to our data, they found that *Gfat1* expression is either absent or very low until 10–14 h AEL (~stages 14–15) and, thereafter, expression is high or very high, with peak expression during the 16–18 h AEL interval (~stage 16).

Our results and the RNAseq data are consistent with the current in situ data for embryonic stages 1–3 indicating maternal deposition of *Gfat2* mRNA, but not of *Gfat1* mRNA [28,45]. Moreover, there is little if any *Gfat1* expression until embryonic stages 13–14 and then very gradually it transitions to a high level throughout the embryo by stages 16–17, during which mRNA appears to be concentrated in dorsal, ventral, and head epidermis and the salivary glands. In distinct contrast, *Gfat2* mRNA is reasonably abundant and ubiquitously distributed in blastoderm embryos (stages 4–6). By embryonic stages 7–8, expression is ubiquitous, with some concentration in various germ layers. The expression then transitions through stages 9–10, during which the mRNA is faintly ubiquitous, but by stages 11–16 it becomes highly abundant and ubiquitous and noticeably concentrated in various tissues and structures.

Protein levels during embryogenesis indicate very low expression from 0–80 min AEL, followed by a very long period of no or extremely low expression from 2 h to ~12 h AEL (embryonic stages 4–16) and then a transition through low expression from 14 to 18 h AEL (stages 16–17), eventually culminating in moderate expression by 20 h AEL [28,43]. The near absence of Gfat1 protein during a long segment of embryogenesis is consistent with the paucity of *Gfat1* mRNA during most of this same period (our data and the RNAseq results). Since there is no clear maternal deposition of *Gfat1* mRNA, it is possible that a small amount of Gfat1 protein is contributed maternally. Proteomic analysis of Gfat2 levels during embryogenesis indicates very low expression from 0–140 min AEL, but thereafter its expression increases transiently to low levels, followed by an increase to moderate expression from 4–14 h AEL, and then ultimately to high expression by 14–20 h AEL. The higher levels of Gfat2 versus Gfat1 protein expression throughout embryogenesis are consistent with the moderately high to high levels of *Gfat2* mRNA throughout embryogenesis described above.

RNAseq analysis during post-embryonic development reveals moderately high or high levels of *Gfat1* expression during most stages, with peak expression during larval stages L2 and 12 h L3, and especially during the 12 h prepupal stage; however, *Gfat1* expression is much lower in adult males and females [28]. The corresponding analysis of *Gfat2* also indicates high expression in L1 and L2, and thereafter predominantly moderately high expression, except for low expression in mid and late pupae. In contrast with *Gfat1*, the expression of *Gfat2* is also moderately high in adult males and females. Interestingly, a very recent study of links between nutrient availability, protein O-GlcNAcylation, and diurnal rhythm in adult flies also found much higher levels of *Gfat2* mRNA versus very low levels of *Gfat1* mRNA [46]. Indeed, based on these and other data, these authors contend that Gfat2 is the primary functional paralogue in adults. Proteomic data indicate high to extremely high *Gfat2* protein expression during all three larval stages and during the white prepupal and days 1 and 3 of pupal development. High levels of the protein were also observed in adult males [28,47]. Corresponding proteomic data for Gfat1 in post-embryonic stages of development are not currently available.

### 3.5. Evidence for Differences in Essential Tissue-Specific Requirements for Gfat1 and Gfat2

Owing to the phenotypic and expression pattern differences observed for *Gfat1* and *Gfat2*, we decided to investigate the question of whether Gfat1 and Gfat2 might have different essential roles in the fly. Thus, we used *Gfat1* and *Gfat2* RNAi transgenes to explore essential tissue-specific requirements for the two versions of the Gfat enzyme (for a description of the RNAi lines used, see Table S1). For the first experiment, we used the *Appl*-*GAL4* pan-neural driver and the *Mef2*-*GAL4* muscle driver. We chose *Appl-GAL4* because of the importance of the HBP in CNS development [48,49,50]. Furthermore, since pilot tests using a previously-generated RNAi transgene showed that RNAi-induced pan-neural and muscle knockdown of *Ogt*/*sxc* was lethal (data not shown), we reasoned that one or both of the Gfat enzymes might be required for HBP generation of the UDP-GlcNAc substrate for essential Ogt catalytic functions (see Figure 1) in these tissues. Therefore, we included RNAi knockdown of *Ogt*/*sxc* in this experiment.

The results of the first experiment are shown in Table 3 and Table 4. As expected, the TRiP RNAi transgene for *Ogt*/*sxc* is lethal when expressed in either the nervous system or muscles. Furthermore, the data also show that one of the *Gfat2* RNAi transgenes is lethal when expressed in both tissues, while the other *Gfat2* RNAi transgene is lethal when expressed in muscles and semi-lethal when expressed in the nervous system. In striking contrast, none of three *Gfat1* RNAi transgenes is lethal when expressed in either the nervous system or muscles. These data support the contention that Gfat2, but not Gfat1, has an essential role in the structure/function of these tissues.

The requirement for proper chitin synthesis in tracheal development in *Drosophila melanogaster* is well documented [49,51,52]. Since *mmy* mutants are defective in chitin production and trachea morphogenesis, we decided to test for possible lethal effects of specific downregulation of *Gfat1* and *Gfat2* in the trachea using *btl*-*GAL4*-driven RNAi transgenes [48,53]. The results of this second experiment are presented in Table 5 and they show that RNAi knockdown of *Gfat1* or *Gfat2* in the trachea is either completely lethal or weakly semi-lethal. These data suggest that both versions of the enzyme have important roles in tracheal development/function.

### 3.6. FISH Localization of the Gfat1 and Gfat2 Genes in Six Different Drosophila Species

**Figure 7:**
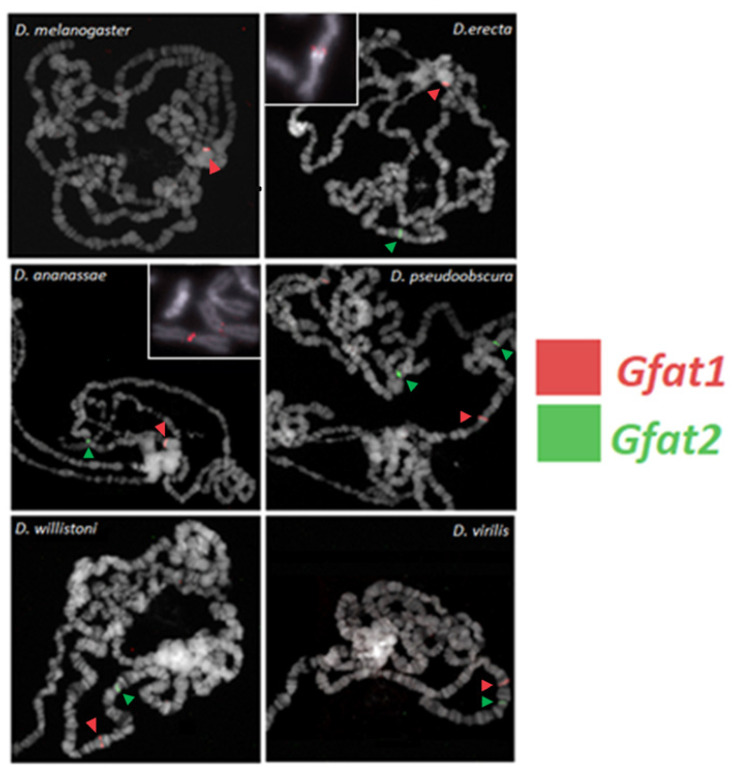
FISH localization of *Gfat1* (red) and *Gfat2* (green) in polytene chromosomes in six and five Drosophila species, respectively. For a description of the methods used, see Pimpinelli et al. [40] Note that *Gfat1* is located within heterochromatin in *D. melanogaster*, *D. erecta*, and *D. ananassae*. This is confirmed for FISH using mitotic chromosomes for *D. erecta* and *D. ananassae* (see the insets). *Gfat1* is euchromatic in *D. pseudoobscura*, *D. willistoni*, and *D. virilis*. *Gfat2* is euchromatic in all species.

It was of interest to investigate the degree to which the heterochromatic location of *Gfat1* was conserved during Drosophila evolution. Thus, we determined the chromosomal locations of the *Gfat1* and *Gfat2* genes in six Drosophila species, two of which are closely related to *D. melanogaster.* The data show that *Gfat2* is located in euchromatin in all six species, whereas *Gfat1* is located in heterochromatin in *D*. *melanogaster* and the two most closely related species, *D*. *erecta* and *D*. *annanasae*, but it is located in euchromatin in the three distantly related species, *D*. *pseudoobscura*, *D*. *virilis*, and *D*. *willistoni* (Figure 7). The most straightforward explanation is that *Gfat1* acquired a position in heterochromatin at or just after the divergence of the melanogaster and obscura groups approximately 25 million years ago (see Figure S3).

### 3.7. Micro-Synteny of Gfat1 and Gfat2 and Flanking Genes

We examined the degree of conservation of the arrangement of genes flanking *Gfat1* and *Gfat2* in the various Drosophila species and the results of this analysis are shown in Table 6 and Table 7. It is noteworthy that the amino acid sequences of *Gfat1* and *Gfat2* are highly conserved across all six species. This is especially the case for *Gfat1*. Furthermore, the same two genes, *CG42402* and *CG40198* (or their orthologs), flank *Gfat1* in all six species; although the 5′ to 3′ orientation of *CG42402* relative to *Gfat1* is highly conserved, that of *CG40198* is variable (Table 6). In addition, for the most part, the distances between the two flanking genes and *Gfat1* are variable, and this is particularly true for *CG40198*. The fact that *Gfat1* and at least two other genes are found together in all six Drosophila species indicates that an entire genomic segment containing Gfat1 was relocated to heterochromatin during the evolutionary divergence of the melanogaster and obscura groups. On the other hand, the identities and relative positions of the genes/orthologs flanking *Gfat2*, *larp* (*CG42551*), and *Moca*-*cyp* (*CG1866*), are somewhat less conserved than those flanking *Gfat1* (Table 7). Thus, while *Moca*-*cyp* is present adjacent to *Gfat2* in all species, its relative position 5′ to *Gfat2* is maintained from *D*. *melanogaster* to *D*. *pseudoobscura* but it is located 3′ to *Gfat2* in *D*. *willistoni* and *D*. *virilis*. Similarly, the position of *larp* 3′ relative to *Gfat2* is conserved to *D*. *pseudoobscura*. However, *larp* is located 5′ to *Gfat2* in *D*. *virilis* and there is no *larp* gene in the immediate vicinity of *Gfat2* in *D*. *willistoni*. Finally, the distances between the flanking genes and *Gfat2* in the six species are variable; however, for the most part, they are shorter than those observed for the genes flanking *Gfat1*.

## 4. Discussion

In this study, we confirm that in *Drosophila melanogaster*, the genes that encode Gfat1 and Gfat2, the rate-limiting enzymes in the HBP are essential, and further, we show that the *Gfat1* gene corresponds to the *zep* locus. The vital nature of the two genes in *Drosophila melanogaster* was reported previously [52,54]. Mattila et al. defined an L1 lethal phase for CRISPR-induced *Gfat2* mutants and Chen et al. reported L1 lethal phases for their CRISPR-induced alleles of both *Gfat1* and *Gfat2*, with some embryonic death [55]. We have found that all *zep* alleles (including the original *zep^LP13^* and newly identified alleles) die as embryos and display the diagnostic blimp embryonic phenotype when homozygous (Figure 3 and data not shown) [47]. In addition, transheterozygotes for some mutant alleles and escapers from RNAi crosses, survive to adulthood at low frequencies and exhibit *Splayed*-like phenotypes. These phenotypes are consistent with the presumptive role of Gfat1 and the HBP in chitin synthesis and thus cuticle formation, as proposed previously [5,24]. Interestingly, peak expression of *Gfat1* mRNA occurs at embryonic stages 16–17, which overlap with the time of synthesis and deposition of the chitinous embryonic cuticle [5,24]. In contrast, our analysis of one of the *Gfat2* deletion mutants revealed a biphasic lethal phase encompassing embryogenesis and early L1, but the mutant embryos showed no cuticle defects. Although there is also a considerable expression of the *Gfat2* mRNA and protein at stages 16–17, it appears that endogenous expression of the *Gfat2* gene is unable to compensate for the effects of *Gfat1* mutants on cuticle deposition. This may be due in part to differences in expression of *Gfat1* versus *Gfat2* in cells that synthesize chitin. However, the data do not rule out the possibility that Gfat2 also contributes to chitin synthesis and cuticle production in the fly.

Our rescue data suggest that the paralogous *Gfat1* and *Gfat2* genes encode functionally equivalent enzymes. However, the different embryonic expression profiles for *Gfat1* and *Gfat2*, coupled with the blimp phenotype exhibited by *Gfat1*/*zep* alleles, suggest that the two genes and their products may have undergone some degree of functional divergence in Drosophila. Our finding of essential requirements for Gfat2, but not for Gfat1, in the nervous system and muscles, provide in vivo support for our contention of functional divergence. We cannot completely rule out the possibility that all three *Gfat1* RNAi lines tested are not sufficiently potent to cause lethality when expressed in these tissues. However, we have observed that the VDRC *Gfat1* RNAi line is lethal in embryogenesis, even when driven by the weak ubiquitous driver, *Armadillo-GAL4* (data not shown), suggesting that this RNAi line is quite potent.

The different essential tissue-specific requirements for Gfat1 and Gfat2 reported here could reflect differences in tissue-specific expression. Although there is considerable overlap with respect to tissue mRNA expression for the two genes, it is noteworthy that modENCODE RNAseq anatomical data show moderately high and low expression, respectively, of *Gfat2* and *Gfat1* mRNA in the central nervous system (CNS) of third instar larvae (http://www.flybase.org (accessed on 29 December 2021)), although comparable anatomical data for Gfat1 and Gfat2 protein expression are as yet unavailable [28]. Interestingly, a study of expression in human tissues has reported complementary, though not entirely mutually exclusive, tissue mRNA expression patterns for *hGfat1* and *hGfat2*. Thus, there is a much higher expression of *hGfat2* in elements of the central nervous system, whereas *hGfat1* shows prominent and abundant expression in a wide variety of tissues, including the pancreas, heart, skeletal muscle, placenta, prostate, testis, etc. [7,56] Very recently it has been determined that protein O-GlcNAcylation in mammalian cardiomyocytes is specifically dependent on Gfat1 but not Gfat2 activity. Indeed, it appears that only Gfat1 is expressed in these cells [57].

Another potential contributory factor to differential tissue-specific activity of Gfat1 versus Gfat2 proteins could involve post-translational modification. For example, it has been shown that the phosphorylation of the PKA site common to both enzymes causes the inhibition of hGfat1 but the activation of hGfat2 [13,58]. In contrast, other studies have reported that this modification causes the activation of both hGfat1 and *Drosophila melanogaster* Gfat1 [5,12]. In addition, there is evidence for the inhibition of hGfat1 by AMP-activated protein kinase (AMPK) phosphorylation at yet another site in the enzyme [59]. Interestingly, the AMPK consensus recognition sequence containing the target serine is conserved in hGfat2 and Drosophila Gfat1 but appears to be absent from Drosophila Gfat2 [5,59]. Thus, in principle, the differential regulation of Gfat1 and Gfat2 by AMPK could contribute to differences in tissue-specific requirements for the activity of these enzymes in Drosophila.

In contrast to our nervous system and muscle results, it appears that both Gfat1 and Gfat2 are essential for tracheal development/function. It has been established that proper tracheal development requires optimal chitin synthesis and deposition [48,51,52]. The fact that there are essential requirements for both enzymes in the trachea suggests that both may contribute to critical chitin synthesis in these cells. However, because of the severity of tracheal phenotypes exhibited by *mmy* mutants, it has been proposed that there are chitin-independent requirements for HBP functions in tracheal development [23,24]. Therefore, it is possible that one of the Gfat enzymes is predominantly or even exclusively responsible for contributing to chitin production in the trachea, whereas the other contributes to other critical functions in these cells.

We have determined that in *D*. *melanogaster* and other members of the *melanogaster* group, the *Gfat1* and *Gfat2* genes are located in heterochromatin and euchromatin, respectively, whereas, in several other more distantly related species, both genes have euchromatic locations. The most straightforward hypothesis is that, in the ancestral Drosophila configuration, both genes were euchromatic and that, during the evolutionary divergence of the melanogaster group, *Gfat1* acquired a heterochromatic location (see Figure S2). It is noteworthy that both genes are located on a single chromosomal element in at least four of the six species (Figure 7). Since *Gfat1* is located in the chromocenter in *D*. *erecta* and *D*. *annanasae*, it is not possible to determine in which chromosome arm they are located, at least from the polytene FISH analysis. Interestingly, both genes are located on chromosome 2 or Muller element E in *D. pseudoobscura*, and this element corresponds to chromosome 3R in *D*. *melanogaster* (gene link at https://blast.ncbi.nlm.nih.gov/Blast.cgi (accessed on 29 December 2021)), suggesting some degree of evolutionary conservation of the element-specific location of the two genes [60,61]. We have also examined the microsynteny of genes that immediately flank *Gfat1* and *Gfat2* in the six species. The same genes/orthologs in the same 5′ and 3′ positions flank *Gfat1* in all six species. By and large, this is also the case for *Gfat2*, although there are some differences for *D*. *willistoni* and *D*. *virilis*. These examples of flanking gene conservation contrast with the pattern for genes that flank another highly conserved gene, *RpL15*, which is located in 3L het in *D*. *melanogaster* (Table S5). In this case, there is a considerable deviation for most species. The significance of conservation of gene arrangements in the vicinities of *Gfat1* and *Gfat2* is unclear but may warrant future investigation.

It is instructive to compare our evolutionary data to those of an earlier seminal analysis of the structure and evolution of *light* and neighbouring heterochromatic genes and their euchromatic orthologues [30]. These workers determined that a large chromosomal segment containing the *light* gene and adjacent genes was juxtaposed to heterochromatin during the evolutionary divergence of the melanogaster subgroup. In contrast, although our results also indicate that a multigene segment that contains the *Gfat1* gene was relocated to heterochromatin, this event occurred during the evolutionary divergence of the melanogaster and obscura groups.

The relationship between *Gfat1* and *Gfat2* and other duplicate gene-pairs in *Drosophila melanogaster* is, as yet, undefined [62]. However, it is noteworthy that there are at least two other Drosophila paralogous gene-pairs for which one gene is heterochromatic and the other is euchromatic: *SNAP25* (3L het)/*SNAP24* (euchromatic) and *spok* (3R het)/*spo* (euchromatic) [63,64,65]. As is true for *Gfat1*/*Gfat2*, these studies show that the two protein products of each of these gene-pairs are functionally equivalent. Interestingly, in all three cases, the two genes exhibit different patterns of mRNA expression [28]. As mentioned, *Gfat2* is expressed at earlier stages and, for the most part, at consistently higher levels than *Gfat1* during embryogenesis. Similarly, *spo* expression occurs during the first half of embryogenesis, whereas *spok* expression begins later in embryogenesis and continues at higher levels in the larval and pupal stages. Finally, *SNAP-24* expression extends throughout embryogenesis at moderately-high/high levels and then decreases somewhat during the larval and pupal stages, whereas *SNAP-25* expression commences rather late in embryogenesis and extends through the larval and pupal stages.

An interesting correlation exists between gene structure and differential expression of the aforementioned gene pairs. Thus, while in each case the heterochromatic paralog either contains multiple introns or one very large intron, the euchromatic paralog either contains a single small intron or is intronless, and this pattern is conserved in the five other Drosophila species included in the current study (*Gfat1*/*Gfat2*: Figures S4 and S5; *SNAP25*/*SNAP24* and *spok*/*spo* data are available in Flybase release FB2017_05 [28]). One possible explanation is that, in each case, the euchromatic paralog has been subject to selective pressure for maintenance of a compact structure that could facilitate rapid and abundant transcription and mRNA processing for earlier developmental requirements. Consistent with this explanation, at least two Drosophila studies have reported enriched identification of intronless and intron-poor genes among genes exhibiting early zygotic expression, particularly those expressed during syncytial stages [66,67]. Moreover, the prevalence of early-expressed zygotic genes with short transcription units is observed for several different species that span the Drosophila phylogeny, suggesting that this pattern is evolutionarily conserved [67].

Future investigations of specific mechanisms of *Gfat1* regulation should provide important insight into the regulation of heterochromatic gene expression. In addition, the question of how *Gfat2* expression is regulated is of considerable interest. Moreover, given the potential links between the HBP and a wide variety of human diseases, further studies of the functional interplay between the Gfat1 and Gfat2 enzymes in flies should prove worthwhile.

## Figures and Tables

**Table 1:** Lethal phase analysis of a *Gfat2* mutation.

Cross	*Gfat2^10A2^*/*Df(3R)BSC460* Offspring		
	Unhatched Embryos	First Instar Larvae	Second Instar Larvae
*Gfat2^10A2^*/*TM3*, *Sb*, *Ser*, *Twi-GFP* x *Df(3R)BSC460/ TM3*, *Sb*, *Ser*, *Twi-GFP*	59	41	0

**Table 2:** Mutant rescue with *Gfat1^+^* and *Gfat2^+^* transgenes.

		Number of Relevant Progeny			
		Mutant/*TM3* or *TM6B*		Mutant/Deficiency or Mutant/Mutant	
Cross	Total	*CyO/Gfat1^+^ or Gfat2^+^*	*Act5C-GAL4/Gfat1^+^ or Gfat2^+^*	*Cy/Gfat1^+^ or Gfat2^+^*	*Act5C-GAL4/Gfat1^+^ or Gfat2^+^*
*UAS*-*Gfat1^+^*/*CyRoi*; *Gfat1^LP13^*/*TM3*, Sb					
X	310	139	120	0	51
*Actin5C-GAL4*/*CyO*; *Df(3R)7B*-*90*, *e*/*TM3*, *Sb*					
*UAS*-*Gfat1^+^*/*CyRoi*; *Gfat2^10A−2^*/*TM3 Ser*					
X	179	115	49	0	15
*Actin5C-GAL4*/*CyO*; *Df(3R)BSC567*/*TM3*, *Ser*					
*UAS*-*Gfat2^+^*/*CyRoi*; *Df(3R)BSC460*/*TM6B*					
X	177	95	50	0	32
*Actin5C-GAL4*/*CyO*; *Gfat2^10A−2^*/*TM3*, *Ser*					
*UAS*-*Gfat2^+^*/*CyRoi*; *Gfat1^z−1904^*/*TM6B*					
X	156	84	54	0	18
*Actin5C-GAL4*/*CyO*; *Gfat1^I400#8^*/*TM3*, Ser					

**Table 3:** Tests for viability effects of *Gfat2*, *Gfat1*, and *Ogt* RNAi knockdown in the larval nervous system at 29 °C using *Appl-GAL4* *.

RNAi	Number of Progeny		Total	Comments on RNAi
	*Gfat* RNAi	Control (Balancer)		
*Gfat2/CyO* (v105129)	123	159	282	male semi-lethality ** frequent unfurled wings
*Gfat2/TM3, Sb* (B34740)	0	218	218	lethal
*Gfat1/TM3, Sb* (HL)	175	111	286	viable
*Gfat1/CyO* (B42892)	258	260	518	viable
*Gfat1* (v24539)	539	-	539	viable
*Ogt/CyO* (B50909)	0	275	275	lethal

* Males heterozygous or homozygous for RNAi transgenes mated to *Appl-GAL4* females. See Table S1 for information about the RNAi lines from stock centres. HL: *2664-1-6M-CH3* line from the Honda lab (see Table S2 for viability data of ubiquitously-expressed RNAi). ** The designation of male semi-lethality (less than 30% of expected) is based on relative viability = the number of observed RNAi males/the number of *CyO* males = 11/44 = 0.25.

**Table 4:** Tests for viability effects of *Gfat2*, *Gfat1*, and *Ogt* knockdown in muscle cells at 29 °C using *Mef2-GAL4* *.

RNAi	Number of Progeny		Total	Comments on RNAi
	*Gfat* RNAi	Control (Balancer)		
*Gfat2/CyO* (v105129)	**0**	148	148	lethal
*Gfat2/TM3, Sb* (B34740)	0	60	60	lethal
*Gfat1/TM3, Sb* (HL)	227	165	392	viable
*Gfat1/CyO* (B42892)	250	252	502	viable
*Gfat1* (v24539)	60	-	60	viable
*Ogt/CyO* (B50909)	0	240	240	lethal

* Males heterozygous or homozygous for RNAi transgenes mated to *Mef2-GAL4* females; genotype of driver: *y w; P{GAL4-Mef2.R}3* (BDSC #27390). See the legend to Table 3 and Table S1 for information about the RNAi lines used.

**Table 5:** Tests for viability effects of *Gfat2* and *Gfat1* RNAi knockdown in the trachea at 30 °C using *breathless-GAL4* *.

Control or RNAi	Number of Progeny		Total	Comments on RNAi
	*Gfat* RNAi	Control (Balancer)		
*w^1118^* control	92	116	208	NA
*Gfat2/CyO* (v105129)	40	132	172	weak semi-lethality **
*Gfat2/TM3, Sb* (B34740)	0	269	269	lethal
*Gfat1/CyO* (B42892)	27	89	116	weak semi-lethality **
*Gfat1* (v24539)	0	202	202	lethal

* Males heterozygous or homozygous for RNAi transgenes were mated to *btl-GAL4*/*CyO* females; genotype of driver: *w; P{w^+mC^ = GAL4-btl.S}2, P{w^+mC^ = UASp-Act5C.T:GFP}2/CyO, P{w^+m^ = lacZ. W^+^}276* (BDSC #8807); see the legend to Table 3 and Table S1 for information about the RNAi lines used. ** The designation of weak semi-lethality (less than 62% of expected) is based on relative viability = number of observed RNAi adults/half the number of *CyO* adults.

**Table 6:** Microsynteny of two genes flanking *Gfat1* or its ortholog in six Drosophila species.

Species and Designation of *Gfat1* Gene	Sequence Homology of Orthologous Gfat1 Proteins Versus *D*. *m*. Gfat1 *	*CG42402* or Ortholog 5′ to *Gfat1* **	5′ to 3′ Orientation Relative to That of *Gfat1*	CG40198 or Ortholog 3′ to *Gfat1*	5′ to 3′ Orientation Relative to That of *Gfat1*
*melanogaster CG12449*	-	yes (22)	same	yes (23)	same
*erecta* *GG12143*	98/98	yes (31)	same	yes (3)	opposite
*ananassae* *GF23135*	95/97	yes (35)	same	yes (12)	same
*pseudoobscura* *GA26267*	97/98	yes (35)	same	yes (6)	opposite
*willistoni* *GK12920*	96/98	yes (38)	same	yes (8)	opposite
*virilis* *GJ24380*	96/98	yes (28)	same	yes (28)	opposite

* percent identity/percent similarity; ** distance from *Gfat1* or ortholog in kbp given in parentheses; data from FB2017_05 release and confirmed via NCBI BLAST.

**Table 7:** Microsynteny of two genes flanking *Gfat2* or its ortholog in six Drosophila species.

Species and Designation of *Gfat2* Gene	Sequence Homology of Orthologous Gfat2 Protein Versus *D*. *m*. Gfat2 *	*Moca*-*cyp* or Ortholog 5′ to *Gfat2* **	5′ to 3′ Orientation Relative to That of *Gfat2*	*larp* or Ortholog 3′ to *Gfat2*	5′ to 3′ Orientation Relative to That of *Gfat2*
*melanogaster CG1345*	-	yes (0.5)	opposite	yes (0.6)	same
*erecta* *GG12070*	98/99	yes (0.4)	opposite	yes (1.3)	same
*ananassae* *GF16128*	95/97	yes (0.9)	opposite	yes (0.7)	same
*pseudoobscura* *GA12297*	92/95	yes (2)	opposite	yes (6)	same
*willistoni* *GK12142*	92/95	located 3′ (0.3)	same	5′ gene: GK12141 (3)	same
*virilis* *GJ22773*	91/94	located 3′ (0.4)	same	located 5′ (0.3)	opposite

* percent identity/percent similarity; ** distance from *Gfat2* or ortholog in kbp given in parentheses; data from FB2017_05 release and confirmed via NCBI BLAST.

## Supplementary Materials

The following are available online at https://www.mdpi.com/article/10.3390/cells11030448/s1. Figure S1: Map of proximal 3R correlating the molecular location of the Gfat1 gene with the genetic position of zep; Figure S2. Splayed phenotype displayed by hs-Gal4/2664-1-5M-CH2 fly raised at 29 °C; Figure S3. Schematic representation of Drosophila evolution over the last 50 million years; Figure S4. Gene architecture of the longest versions of Gfat1 in six species of Drosophila; Figure S5. Gene architecture of Gfat2 in six species of Drosophila; Table S1. RNAi strains from Drosophila stock centres used in the present study; Table S2. Tests for viability effects of Gfat1 RNAi transgenes when driven ubiquitously; Table S3. Primers used for the sequencing analysis of zep alleles; Table S4. qPCR primer sequences; Table S5. Microsynteny of two genes flanking RpL15 or its orthologue in six Drosophila species.
