# Supplementary material for: Characterization of Gfat1 (zeppelin) and Gfat2, Essential Paralogous Genes Which Encode the Enzymes That Catalyze the Rate-Limiting Step in the Hexosamine Biosynthetic Pathway in Drosophila melanogaster
Source: Cells. 2022 Jan 27;11(3):448. doi: 10.3390/cells11030448 (PMC8834284; doi:10.3390/cells11030448)
Supplement: Supplementary file 1 [file cells-11-00448-s001.zip › cells-1558320-supplementary.pdf]

cells MDPI

Supplementary

Characterization of *Gfat1* (*zeppelin*) and *Gfat2*, Essential Paralogous Genes Which Encode the Enzymes That Catalyze the Rate-Limiting Step in the Hexosamine Biosynthetic Pathway in *Drosophila melanogaster*

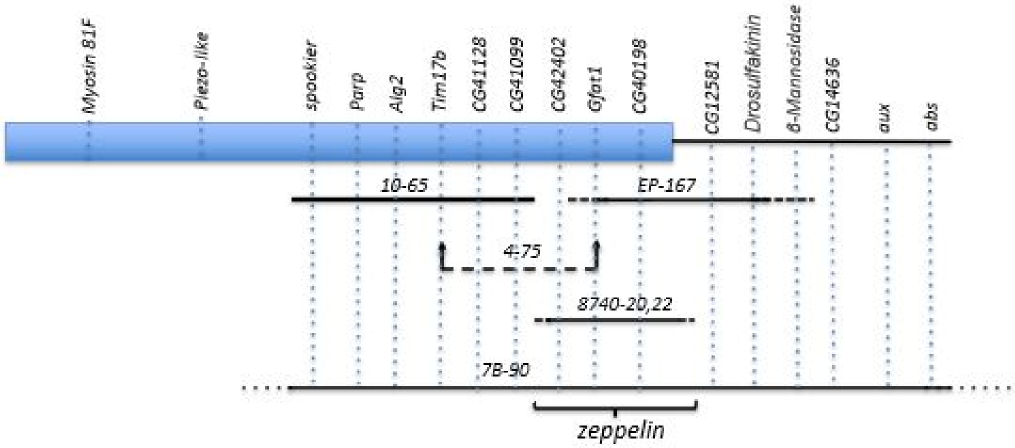

Figure S1. Map of proximal 3R correlating the molecular location of the *Gfat1* gene with the genetic position of *zep*. The extents of the various rearrangements used are shown below. *In(3R)4-75* is a multi-break inversion, whereas the other rearrangements are deletions in proximal 3R [35, 35, 41].

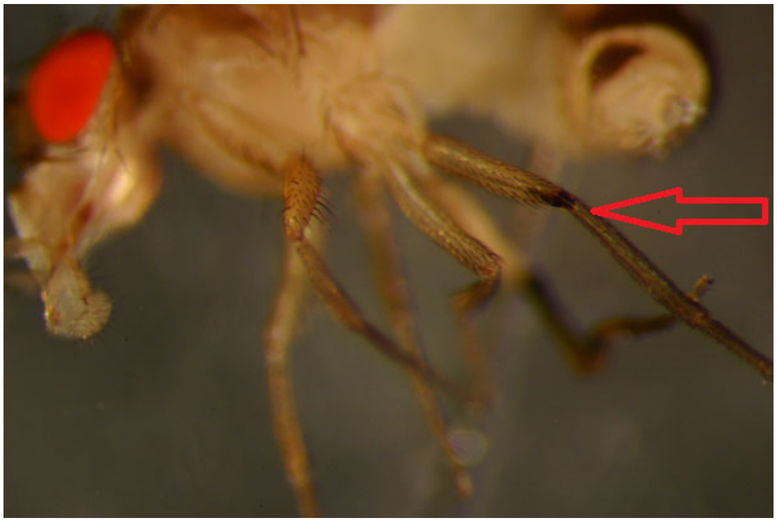

Figure S2. *Splayed*-like phenotype displayed by hs-*GAL4*/*2664-1-5M-CH2* fly raised at 29°C. Note the melanin deposit on the leg as indicated by the red arrow.

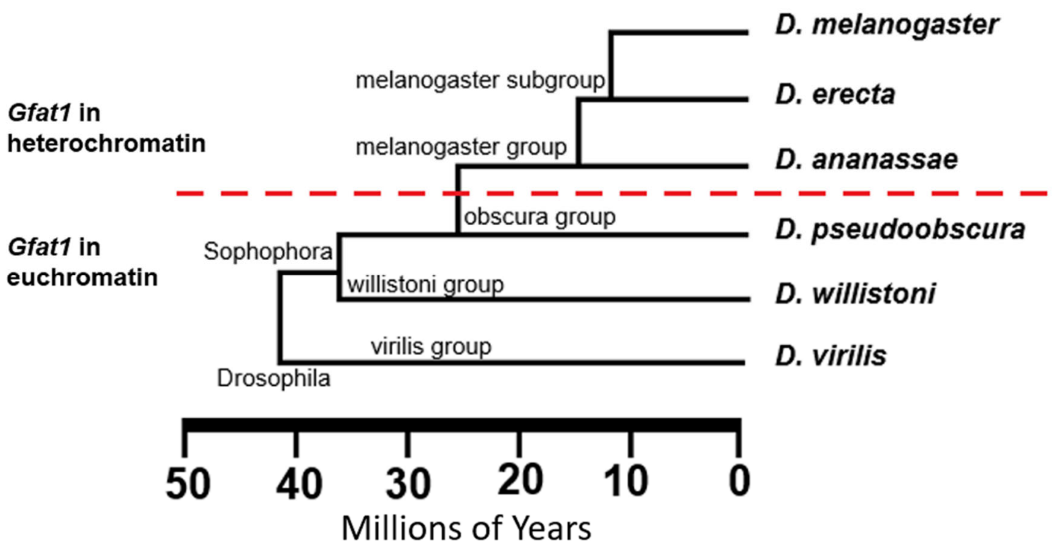

Figure S3. Schematic representation of Drosophila evolution over the last 50 million years. The dashed red line separates the species group in which the *Gfat1* gene is located in heterochromatin (melanogaster group) from that in which the gene is located in euchromatin (obscura group).

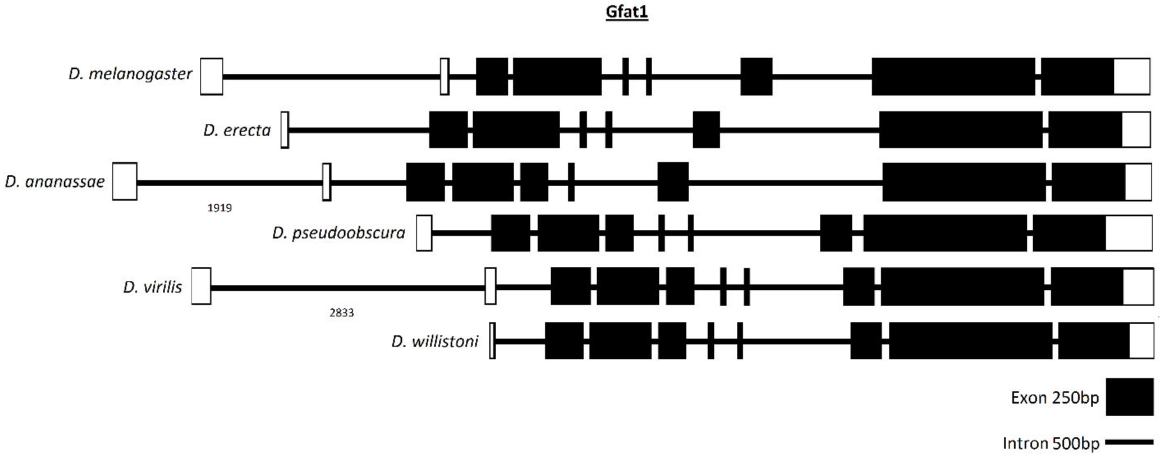

Figure S4. Gene architecture of the longest versions of *Gfat1* in six species of Drosophila. Translated exons are represented by black boxes. 5′ and 3′ untranslated regions (UTRs) are represented by white boxes. Introns are represented by black lines. Data from FB2017_05 release.

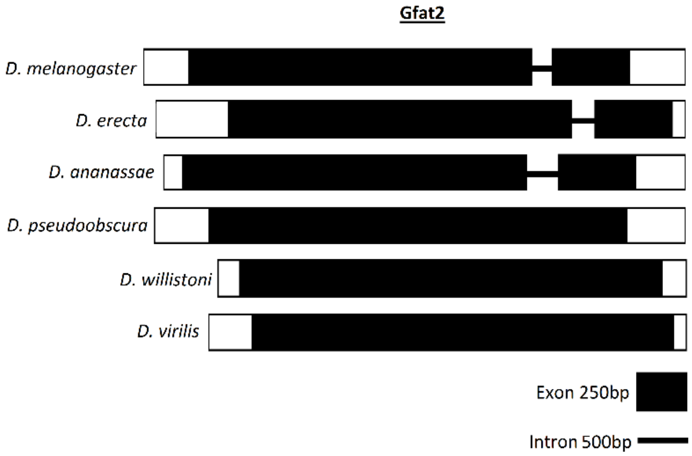

Figure S5. Gene architecture of *Gfat2* in six species of Drosophila. Translated exons are represented by black boxes. 5′ and 3′ UTRs are represented by white boxes. Introns are represented by black lines. Data from FB2017_05 release.

Table S1. RNAi strains from Drosophila stock centres used in the present study.

Stock Number and Genotype	Origin	Gene	Designation
v24539: *w^{1118}; P{GD7732}v24539*	VDRC	*Gfat1*	v24539
42892: y sc* *v sev^{21}; P{TRiP.HMS02585}attP40*	BDSC	*Gfat1*	B42892
v105129: *P{KK101769}VIE-260B*	VDRC	*Gfat2*	V105129
34740: *y sc* v sev^{21}; P{TRiP.HMS01220}attP2/TM3, Sb*	BDSC	*Gfat2*	B34740
50909: *y v; P{TRiP.HMJ03131}attP40*	BDSC	*Ogt*	B50909

Table S2. Tests for viability effects of *Gfat1* RNAi transgenes when driven ubiquitously. Chromosome 2 insertions crossed with *Act5C-GAL4/Cy0;*. Chromosome 3 insertions crossed with *Tub-GAL4/TM3, Sb.*

Transgene and Location	***CyO* or *TM3, Sb* Progeny**	RNAi with Driver
Chromosome 2		
2664-1-2M-CH2	100	0
2664-1-5M-CH2	55	0
2664-1-9M-CH2	42	0
Chromosome 3		
2664-1-6M-CH3	58	0
2664-1-7M-CH3	47	0
2664-1-8M-CH3	40	0

Table S3. Primers used for the sequencing analysis of zep alleles. Primers span the entire coding sequence of the *Gfat1* gene (including exon/intron splice junctions). They are depicted 5′ to 3′ from left to right. Gfat1 sequencing primers.

**GFP-F caagagtgccatgcccgaag**	**GFP-R gacagggccatcgccaattg**
Grp84-01 acgcttctcgctgatggac	Grp84-02 tccagtaactggattgagt
Gfat1-1F tcttggcttgtctgcgtgctacc	Gfat1-1B aaccgagttttggctgctgat
Gfat1-2F tcgcgttcgcattttcacaag	Gfat1-13 ccaacgtgggtcaggacgggttc
Gfat1-4F actacaaggttcaacattctg	Gfat1-4B gggaaatgaattgggaggtgt
Gfat1-5F ctgatggccttacgttactgt	Gfat1-5B atgtgtccaagcaatgtgatg
Gfat1-15 ctcgcgcgcaatcaacatacgatt	Gfat1-6B caagtgtaagatggtaggtgc
Gfat1-10F gtaagcacagcaatggtt	Gfat1-14F gccacttgcctagaaggtgga
Gfat1-24R cggcaatatgataagacagtagtagc	

Table S4. qPCR primer sequences. Gene Amplified Primer and Probe Sequences.

RpL32	**Forward primer 5′- GTCGGATCGATATGCTAAGCTG -3′**
	Reverse primer 5′- CAGATACTGTCCCTTGAAGCG -3′<br>Probe 5′-/56-FAM/ TCGATATGC /ZEN/ TAAGCTGTCGGCACAAA /3IABkFq/-3′
Gfat1	Forward primer 5′- ACCTATACCAACACAAGTCGC -3′<br>Reverse primer 5′- CTTCAATTCACCGGCCATG -3′<br>Probe 5′-56FAM/TGCCTAGAA/ZEN/GGTGCATTGAAAGTCAAAGAG /3IABkFQ/ -3′
Gfat2	Forward primer 5′- TTGCCAAGGAGTTGTACGAG -3′<br>Reverse primer 5′- TCCGTGTTTCAGTTCTCCAG -3′<br>Probe 5′-/56-FAM/ AGTTGAAGC /ZEN/ CCCTGCCCATAATGA /3IABkFQ/-3′

Table S5. Microsynteny of two genes flanking RpL15 or its orthologue in six Drosophila species. Data from Schulze et al., 2005 and the FB2017_05 release; note that RpL15 is located in heterochromatin in D. virilis, but in euchromatin in D. pseudoobscura (Schulze et al., 2005); sequence identity of orthologous RpL15 proteins versus D.melanogaster. RpL15 range from 96% to 100%. .

**Species and Designation of RpL15 gene**	**rad21 or Ortholog 5′ to RpL15**	**5′ to 3′ Orientation Relative to RpL15**	**Dbp80 or Ortholog 3′ to RpL15**	**5′ to 3′ Orientation relative to RpL15**
melanogaster CG17420	yes	same	yes	same
erecta GG163267	GG13128	opposite	yes	same
ananassae GF20530	yes	opposite	GF27128	opposite
pseudoobscura GA22222	GA31871	same	yes	opposite
willistoni GK12236	GK10847	opposite	GK10846	opposite
virilis GJ22514	GJ22512	opposite	GJ22515	same
